# Modified poly-L-lysine for use as a clearing agent in pretargeted radioimmunotherapy

**DOI:** 10.1186/s41181-024-00307-6

**Published:** 2024-11-13

**Authors:** Chiara Timperanza, Anna Gustafsson-Lutz, Tom Bäck, Damian J. Green, Sture Lindegren, Emma Aneheim

**Affiliations:** 1 Department of Medical Radiation Sciences, Institute of Clinical Sciences, Sahlgrenska Academy, University of Gothenburg, Gothenburg, Sweden; 2 Fred Hutchinson Cancer Center, Seattle, WA USA; 3 University of Washington, Seattle, WA USA; 4 Department of Oncology, Region Västra Götaland, Sahlgrenska University Hospital, Gothenburg, Sweden

**Keywords:** Pretargeted radioimmunotherapy, Pretargeting, Clearing agent, Poly-L-lysine, Streptavidin/avidin–biotin, Tetrazine-Transcyclooctene/TCO, Click chemistry

## Abstract

**Background:**

Pretargeted radioimmunotherapy of cancer has the potential to increase tumor specific uptake of activity when compared with conventional radioimmunotherapy. This is especially true in radioimmunotherapy with nuclides that exhibit a relatively short half-life. When administering antibody-based pretargeting molecules systemically, the antibodies often show a relatively slow clearance from the blood. Therefore, the use of a clearing agent is advantageous to remove unbound pretargeting molecules from the circulation, facilitating a reduction in the nonspecific radiation exposure to normal tissue while maximizing the dose delivered to the tumors.

**Results:**

In the current study, two types of poly-L-lysine based clearing agents were produced for two different pretargeting systems: (strept)avidin/biotin and Tetrazine/Transcyclooctene. Poly-L-lysine was used as scaffold for production of clearing agents. The polymer is available in multiple sizes and can readily be modified with several functional groups, allowing different pretargeting strategies to be used. In vivo evaluation of the biotin-functionalized poly-L-lysine clearing agent, 110 repeating units, resulted in a decrease in blood concentration of the Iodine-125 labeled pretargeting agent of 50%, circa 23 h after injection, compared to controls. Two sizes, 68 and 143 repeating units, of the tetrazine-functionalized poly-L-lysine clearing agent were also evaluated, which at 23 h after injection decreased the blood concentration of the Iodine-125 labeled pretargeting agent to 58 and 38% respectively.

**Conclusion:**

The straightforward synthesis of poly-L-lysine based clearing agents makes kit preparation possible and these agents show good potential for further evaluation, especially within the Tetrazine/Transcyclooctene pretargeting system where no liver or kidney accumulation was observed.

## Background

Treatment options using radioimmunotherapy are currently limited due to the slow pharmacokinetics of antibodies (Myrhammar et al. 2020). To circumvent these problems, pretargeted radioimmunotherapy (PRIT) has been used to achieve a higher tumor activity uptake relative to normal tissue than the usual conventional radioimmunotherapy (Frost et al. 2011; Sharkey et al. 2012; Frampas et al. 2013). In PRIT, the therapeutic process is divided into two or three steps to improve distribution of the radioactivity. First, a pretargeting agent is introduced in the form of tumor-specific antibodies modified with certain active groups displaying high affinity towards a smaller payload-carrying effector molecule. The effector is generally a small molecule with fast circulatory distribution and blood clearance to which an active group can be conjugated with a high affinity for the active group on the pretargeting agent (Frost et al. 2011; Park et al. 2010; Khaw et al. 2006).

After administration, the pretargeting agent is allowed to distribute and accumulate at the tumor site for several hours until a maximum tumor-bound fraction is reached. Due to the slow clearance of antibodies in vivo, the concentration of unbound antibodies in the blood perseveres. To enhance the Tumor-To-Blood (TTB) ratio of the pretargeting molecules, a clearing agent (CA) that increase removal of the pretargeting agent from the blood can be applied (Bauer 2023). Therefore, a CA can be used as a means to maximize the therapeutic window in PRIT (Press et al. 2001; Liu et al. 2010; Rossin et al. 2013). After the clearing agent has removed the unbound pretargeting agent from the blood, the radiolabeled effector molecule can be administered. (Frost et al. 2011; Park et al. 2010; Khaw et al. 2006; Pagel et al. 2011).

Historically, one of the most studied pretargeting systems exploits the strong affinity between biotin (vitamin H) and avidin or streptavidin. Avidin and streptavidin are large proteins (approximately 66 kDa and 60 kDa, respectively) capable of binding four biotin molecules per unit (Frost et al. 2010). The exceptional binding strength between avidin and biotin is among the most potent non-covalent biological interactions known. Streptavidin is similar to avidin with the main functional difference being that streptavidin does not contain any galactose group. This leads to longer retention of streptavidin in circulation compared to avidin. On the other hand, avidin will rapidly be sequestered by the liver after intravenous (i.v) administration. While the accumulation of streptavidin in the liver is lower than for avidin, the immunogenicity is higher (Pagel et al. 2009).

Since the introduction of pretargeting, diverse clearing agent methods have emerged. Clinical approaches have included avidin, carbohydrate, and albumin-based CAs (Staudt and Herth 2023). The majority of these CAs relied on the (strept)avidin–biotin interaction, however, immunogenic reactions have reduced their usage. The immune response can also reduce the effectiveness of the CA upon repeated administrations, hampering the use in a fractionated dose delivery system (Marshall et al. 1996). More contemporary pretargeting methods, like tetrazine (Tz) and trans-cyclooctene (TCO) ligation, are gradually being tested (Staudt and Herth 2023) but so far, no golden standard or off-the-shelf clearing agent exist for any type of pretargeting system. Based on the Inverse Electron Demand Diels Alder (IEDDA) reaction, the interaction between Tz and TCO belongs to the class of reactions termed click chemistry. Click chemistry is used to join two molecular entities and is usually associated with high yields, minimum side products, efficient reaction rates, and tolerance to a variety of functional groups and solvents (Bauer 2023; Bird et al. 2021).

When using (strept)avidin-conjugated pretargeting molecules, the CA would contain biotin to bind to one of the four biotin-binding sites of (strept)avidin, while in the case of click chemistry the CA would contain tetrazine to bind to the TCO moiety on the antibody pretargeting molecule. Furthermore, galactose could be incorporated in the CA to direct the CA-pretargeting molecule complex to the Ashwell receptors of the hepatocytes in the liver (Breitz et al. 2000), where the pretargeting molecule is degraded and no longer can influence the effector molecule uptake.

ɛ-poly-L-lysine is a non-inert, naturally occurring polymer with low toxicity, suitable for use in e.g. drug delivery systems and biomaterials (Patil 2021). In previous studies poly-L-lysine based effector molecules for conjugation with the alpha emitter astatine-211 have been developed and evaluated using both the (strept)avidin/biotin and the click chemistry pretargeting systems (Rondon et al. 2017; Lindegren et al. 2002; Timperanza, et al. 2023). In this proof-of-concept work the same approach was used to construct and investigate clearing agents based on the versatile and easily modified poly-L-lysine backbone for the two different pretargeting systems in vivo. Due to difficulties in synthesis an already well-established pretargeting agent was used for the (strept)avidin/biotin system (Hylarides et al. 2001), whereas the pretargeting agent for the Tetrazine/TCO system was synthesized in-house.

## Methods and materials

This study aims to investigate poly-L-lysine based clearing agents for use within two different pretargeting strategies, the (strept)avidin/biotin system and the Tetrazine/TCO, or click chemistry, system.

The in vivo experiments were carried out in strict accordance with the Swedish national legislation on protection of laboratory animals. The animal study protocol was approved by the Committee on the Ethics of Animal Experiments at the University of Gothenburg (Idnr: 002138 May 15, 2019). All aqueous solutions were prepared using ultrapure MilliQ water (> 18.2 MΩ*cm) and all organic reagents were of analytical grade or better. All the chemicals used in the study were purchased from Sigma-Aldrich Sweden AB unless otherwise stated. Size-exclusion chromatography was performed using NAP-5 and NAP-10 columns (Cytiva, UK). The radioactivity measurements were conducted using a NaI(Tl) γ counter (Wizard 1480, Perkin Elmer, USA).

### Pretargeting molecules

For the (strept)avidin/biotin pretargeting system the pretargeting molecule (MAb-SA) was provided by Dr. Oliver W. Press, Fred Hutchinson Cancer Research Center (Seattle, WA, USA) and synthesized as previously described (Hylarides et al. 2001) using 1F5, which is a monoclonal antibody (mAb) targeting the CD20 protein. CD20 is an activated-glycosylated phosphoprotein, which is highly expressed in B-cell lymphomas and can therefore be used in RIT and PRIT of non-Hodgkin’s lymphoma (Pagel et al. 2009; Pagel et al. 2007; Pantelias et al. 2007).

For the click chemistry pretargeting system, synthesis of the pretargeting agent is much more straightforward and was done in-house. Trastuzumab (TMAb; Herceptin®) was obtained from Apoteket AB, Sahlgrenska University Hospital (Gothenburg, Sweden) and was used to synthesize the TMAb-TCO pretargeting agent. Trastuzumab is a mAb that specifically targets HER2 + cancer cells (Myrhammar et al. 2020) and can be used in RIT and PRIT of several cancer types, e.g. breast cancer, ovarian cancer, and stomach cancer (Heyerdahl et al. 2011; Keinanen et al. 2017).

#### Synthesis of TMAb-TCO pretargeting agent

The TMAb-TCO pretargeting agent was synthesized by conjugating trans-4-Cycloocten-1-yl 2,5-dioxo-1-pyrrolidinylcarbonate-N-hydroxysuccinimide (TCO-NHS; Click Chemistry Tools, Scottsdale, AZ) to Trastuzumab. Before conjugation the antibody was buffer exchanged twice, first on a NAP-5 and then on a NAP-10 size exclusion column. The purified antibody was eluted in 0.2 M carbonate buffer at pH 8.5 to a concentration of 9.3 mg/mL. TCO-NHS was dissolved in dimethylformamide (DMF) to a concentration of 10 mg/mL. Then, 33 µL of TCO-NHS was added to 1000 µL of the MAb solution, corresponding to a molar excess of 20 times, and the mixture was left to stir protected from light for 2 h at room temperature (RT). Finally, the conjugated antibody was purified on an NAP-5 column and the product was eluted in phosphate buffered saline (PBS). This standard procedure normally leads to the conjugation of between 2 and 6 TCO molecules per antibody (Rondon et al. 2017; Qiu et al. 2019; Sarrett et al. 2021; Summer, et al. 2018).

### Synthesis of the biotin-based clearing agent

**Fig. 1:**
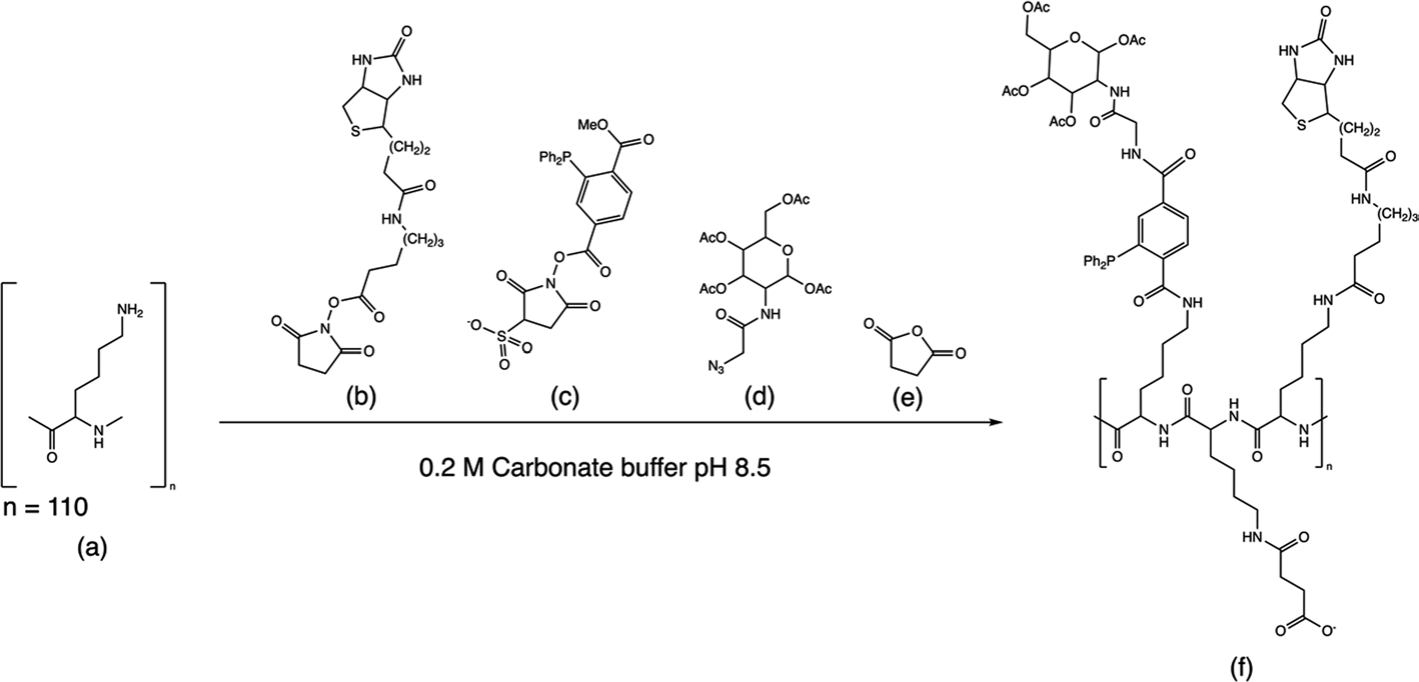
Synthesis of the biotin based clearing agent (biotin-PL-CA). Poly-L-lysine reacts with **a** Succinimidyl-6-(biotinamido)hexanoate (**b**), sulfo-NHS-phosphine (**c**), and tetraacylated *N*-azidoacetylgalactosamine (**d**). The molecule is also charge-modified with succinic anhydride (**e**) to finalize a clearing agent ready for intravenous injection (i.v.) (**f**)

Poly-L-lysine (PL) consisting of approximately 110 (16 kDa) lysine residues was dissolved to a concentration of 5 mg/ml (0.31 mmol) in 0.2 M carbonate buffer (pH ~ 8.5). Succinimidyl-6-(biotinamido)hexanoate (Thermo Fisher Scientific Inc.) in dimethyl sulfoxide (DMSO; 12 mg/ml; 0.026 M) was first added to the PL in a tenfold molar excess and the reaction was stirred for 30 min at RT. A solution of 0.01 M of sulfo-NHS-phosphine (Pierce Biotechnology, Rockford, IL, USA) in DMSO was then added to the biotin-PL in a 20-fold molar excess and the reaction let to stir for 30 min at RT. Subsequently, 0.01 M of tetraacylated *N*-azidoacetylgalactosamine (Pierce Biotechnology) in DMSO was added to the reaction in a 30-fold molar excess, and the mixture was stirred overnight at RT. The PL was thereafter charge-modified with succinic anhydride, which was added in a ~ 50-fold molar excess relative to the ε-amino groups, to form the biotin based clearing agent (biotin-PL-CA) . The charge modification was allowed to proceed for 30 min while the pH was kept constant at ~ 8.5 by repeated addition of 1 M Na_2_CO_3_, Fig. 1.

### Synthesis of the Tetrazine-based clearing agent

**Fig. 2:**
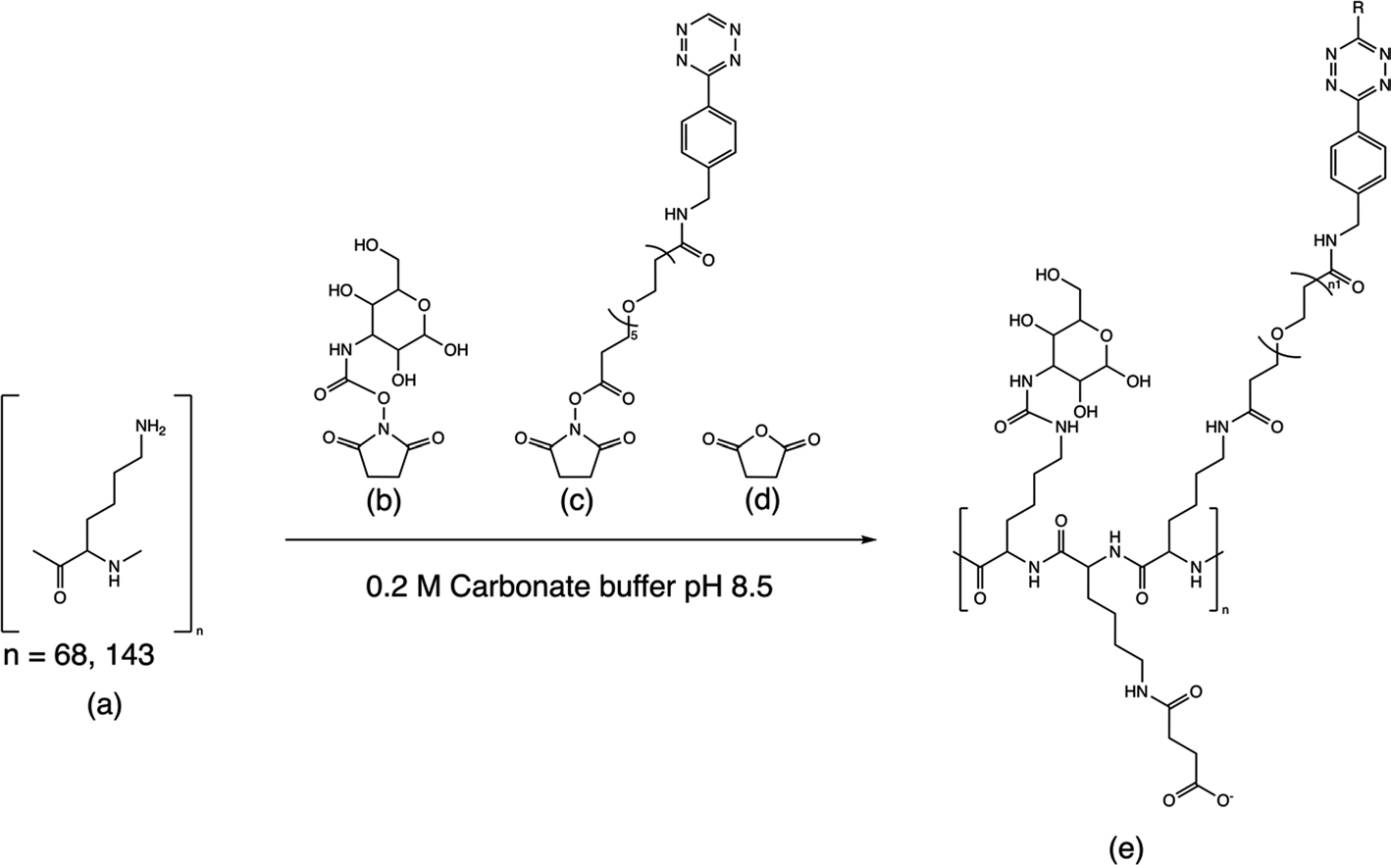
Synthesis of the tetrazine based clearing agent (Tz-PL-CA). Poly-L-Lysine reacts with (**a**) galactose-N-hydroxysuccinimide ester (**b**), Tetrazine-N-hydroxysuccinimide ester (**c**), and finally with succinic anhydride (**d**) for charge modification to give the CA ready for intravenous injection (i.v.) (**e**)

To synthesize the Tetrazine-based clearing agent (Tz-PL-CA), two PL chain lengths were used. PL consisting of 143 (21 kDa) and 68 (10 kDa) lysine repeating units were dissolved in 0.2 M carbonate buffer (pH 8.5) at a concentration of 4 mg/mL. Galactose-N-hydroxysuccinimide ester (Galactose-NHS, Xi'an Ruixi Biological Technology Co.Ltd Xi'an city,Shaanxi Province, China) was dissolved in DMSO to a concentration of ~ 50 mg/ml and added to the PL solution. The mixture was stirred at RT for 30 min. Then, Tetrazine-N-hydroxysuccinimide ester (Tz-NHS) was dissolved in DMSO to a concentration of 200 mg/mL and added to the reaction mixture, in 4.8 molar excess, and the reaction was run at RT for another 30 min. Solid succinic anhydride was added at 4 times molar excess relative to the ε-amino groups for charge modification, Fig. 2. Similarly, as above described, the pH was kept constant at ~ 8.5 by repeated addition of 1 M Na_2_CO_3_. After 30 min, the resulting Tz-PL-CA was purified by size exclusion chromatography using a NAP-10 column. The product was eluted in PBS at pH 7.4. The corresponding PL and reagent masses are listed in Table 1.

**Table 1:** Poly-L-lysine conjugation conditions for the tetrazine-based clearing agent (Tz-PL-CA)

Poly-L-Lysine			Galactose-NHS ester		Tetrazine		Succinic Anhydride	
(kDa)	(mg)	(nmol)	(mg)	(µmol)	(mg)	(µmol)	(mg)	(µmol)
21	1.8	78	~ 1.3	~ 1.1	1.1	1.8	4.8	48
10	1.6	160	~ 1	~ 0.8	0.5	0.8	4.3	43

### ^125^I-labeling of the pretargeting agents

The pretargeting molecules were labeled with ^125^I to be able to monitor their concentration in the blood circulation. A mild solid-phase iodination method was used in order to avoid any potential damage to the pretargeting antibody (Protocols and Handbook et al. 1996). Briefly, an Eppendorf tube was coated with 1 mg/ml 1,3,4,6-tetrachloro-3α,6α-diphenyl glycoluril (Pierce Iodination reagent, previously called “IODO-GEN”; Thermo Fisher Scientific Inc.) in chloroform, and the chloroform was allowed to evaporate with nitrogen gas. To the Eppendorf tube, 0.1 M NaH_2_PO_4_/0.2 M Na_2_HPO_4_, the MAbs, and ^125^I (PerkinElmer, Waltham, MA, USA) were added and allowed to incubate for 2 min to iodinate the protein tyrosine residues. Subsequently, the ^125^I-labeled pretargeting molecules were purified using a NAP-5 size exclusion column.

#### Radiochemical purity

The radiochemical purity of the ^125^I-labeled MAbs was analyzed in triplets by trichloroacetic acid precipitation (TCA) (Frost et al. 2010). Approximately 1–1.5 kBq of ^125^I-MAbs was added to a test tube containing 200 µL of 1% bovine serum albumin (BSA) in PBS, pH 7.4, and the total sample activity was measured before adding 500 µL of TCA for instant precipitation of the protein. The mixture was then centrifuged for 2 min after which an aliquot sample of the supernatant was separated from the pellets, the non-bound activity was measured from the aliqoute, and the radiochemical purity calculated.

### Animals experiments

Healthy female BALB/c mice (Charles River Laboratories International, Wilmington, MA, USA) were used in the in vivo evaluation of the PL-based clearing agents. The mice were housed at 22 °C in 50–60% humidity with a light/dark cycle of 12 h. The animals were kept under pathogen-free conditions. The mice administered with biotin-PL-CA were fed biotin-free food pellets. All the mice had water ad libitum during the 4 days before experiment start. The dose of the CAs was chosen to exceed the amount of circulating PRIT-agent, which in turn was determined by estimating an excess of the amount of the available binding sites on a prospective tumor. All animal experiments were approved by the Ethics Committee at the University of Gothenburg (Gothenburg, Sweden).

#### In vivo evaluation of the biotin-based clearing agent

Six mice were divided into two groups of three individuals each. All mice were intravenously (i.v.) administered with 300 µg (1.4 nmol) of ^125^I-labeled 1F5-SA. In total, 400 µg (1.9 nmol) of HB8181 hybridoma (immunoglobulin G_2a_ isotype control; American Type Culture Collection, Manassas, VA, USA) was co-injected to prevent non-specific binding to Fc receptors in the spleen and other organs. The biotin-PL-CA (5.8 nmol) was injected in three mice 23 h after administration of the iodinated 1F5-SA, the other three mice were used as controls and therefore did not receive any CA. The timepoint was chosen to be experimentally convenient. Blood samples were taken from the tail vein of the mice in both groups at 4 h, 23 h, 23.5 h, 24 h, 25 h, 27.5 h, and 46 h after injection of ^125^I-1F5-SA, and the percentage of the injected activity per gram (% IA/g) in the blood of the mice was evaluated. Activity uptake in the liver and kidneys was measured 68 h after administration of the ^125^I-1F5-SA through biodistribution.

#### In vivo* evaluation of the Tetrazine-based clearing agents*

Eleven mice were divided into two groups of four individuals each for the large Tz-PL-CA (137 µg; 6.5 nmol) and the small Tz-PL-CA (65 µg; 6.5 nmol) and three mice were used as controls. All mice were intraperitoneally (i.p.) administered with ~ 200 µg (1.3 nmol) of ^125^I-labeled TMAb-TCO. 23 h after administration of the iodinated trastuzumab, the poly-L-lysine-based CAs were injected. The three control mice did not receive any CA. Blood samples were taken from the tail vein of the mice in all groups at 2.5 h, 22.5 h, 24.5 h, 25 h, 30 h, and 45 h after injection of the trastuzumab, and the percentage of the injected activity per gram (% IA/g) in the blood of the mice was evaluated. Activity uptake in the liver and kidneys was measured 48 h after administration of the ^125^I-trastuzumab-TCO.

### Statistical analysis

In this study unpaired t-tests were performed in order to assess the statistical difference in the mean between controls and the different clearing agents as well as between the two Tz-PL-CA sizes.

## Results

### The (strept)avidin/biotin pretargeting system

**Fig. 3:**
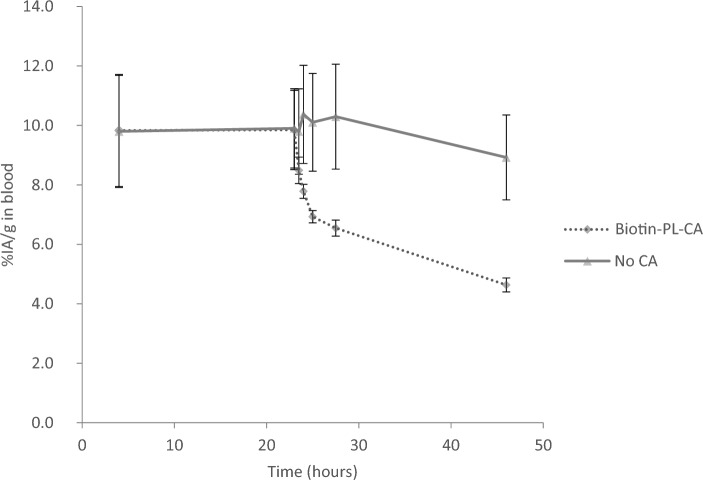
Effects of a biotin-based clearing agent (biotin-PL-CA) on circulating ^125^I-1F5-Streptavidin. The line plot shows the blood profile of the pretargeting agent with and without clearing agent (CA), reported as the percentage of injected activity per gram (%IA/g). The mean and standard error of the mean (SEM; error bars) are depicted

The pretargeting agent ^125^I-1F5-Streptavidin (^125^I-1F5-SA) (Hylarides et al. 2001) was used to evaluate the clearing performance of the synthesized biotin-poly-L-lysine clearing agent (biotin-PL-CA), 110 lysine repeating units. The blood content of ^125^I-1F5-SA over time (% IA/g) showed an immediate drop in concentration after administration of the biotin-based CA. After 4.5 h, the concentration of ^125^I-1F5-SA in the blood of the mice receiving CA was approximately 60% of ^125^I-1F5-SA levels in control animals (*p* = 0.0984). After 23 h, ^125^I-1F5-SA levels in mice receiving CA had dropped further to approximately 50% of that in the control group (*p* = 0.0412), Fig. 3.

**Fig. 4:**
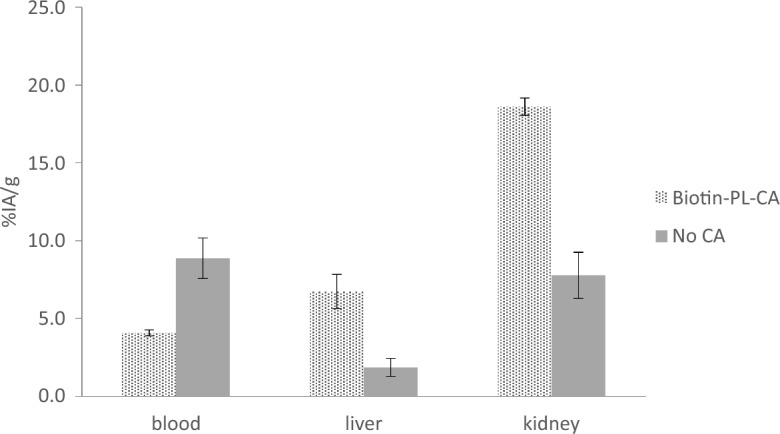
Organ uptake in mice of ^125^I-1F5-steptavidin pretargeting agent or fragments/conjugates thereof, 68 h after administration of a biotin-based clearing agent (biotin-PL-CA) compared with reference animals not receiving any clearing agent (CA). The mean and standard error of the mean (SEM; error bars) are depicted

Uptake of radioactivity in the liver and kidney was measured 68 h after administration of the ^125^I-1F5-SA to follow the clearance route and estimate the level of decomposition of ^125^I-1F5-SA with and without CA. The blood concentration of ^125^I-1F5-SA in mice receiving biotin-PL-CA was still approximately 50% compared with the control group (*p* = 0.0198), Fig. 4. On the other hand, uptake of ^125^I-1F5-SA in the liver was approximately 4 times higher in the mice injected with CA (*p* = 0.0173). In line with the liver uptake, the kidney uptake of activity was more than 2 times higher in the mice receiving CA (*p* = 0.0025).

### The Tetrazine/Transcyclooctene pretargeting system

**Fig. 5:**
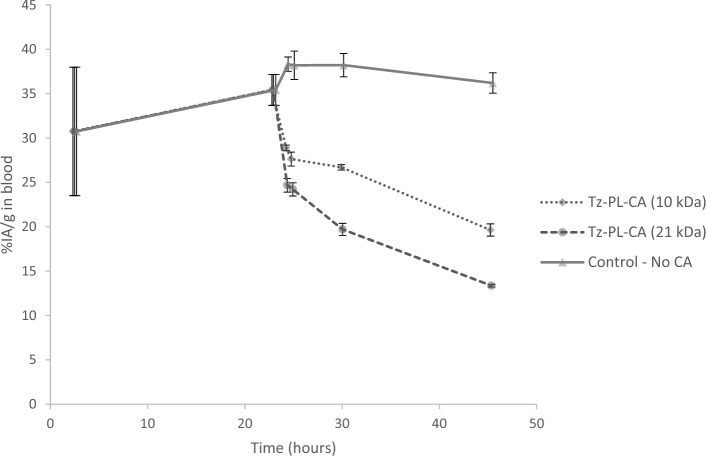
Effects of a tetrazine-based clearing agent (Tz-PL-CA) on circulating ^125^I-trastuzumab transcyclooctene molecules. The line plot shows the blood profile of the pretargeting agent with and without clearing agent (CA), reported as the percentage of injected activity per gram (%IA/g). The mean and standard error of the mean (SEM; error bars) are depicted

An in-house synthesized and Iodine-125 labelled pretargeting agent consisting of TCO substituted Trastuzumab (^125^I-TMAb-TCO), was used to evaluate two sizes of tetrazine substituted, glucosylated and succinilated poly-L-lysine clearing agents. Poly-L-lysine of, 21 and 10 kDa, 143 and 68 repeating lysine units, were evaluated for the Tz-PL-CA. After 7 h the blood content of ^125^I-TMAb-TCO dropped to 56% after the administration of the larger Tz-PL-CA (143) and to 79% after the administration of the smaller, Tz-PL-CA (68), compared to controls (*p* < 0.0001 for both sizes). After 23 h, the ^125^I-TMAb-TCO levels in mice receiving the CAs decreased further to 38% for the large PL and 58% for the small PL (*p* < 0.0001 for both sizes), Fig. 5. These results also showed that there was a significant difference between the two Tz-PL-CA sizes (*p* < 0.0001), with the larger being more effective in the clearing of the PRIT-agent.

**Fig. 6:**
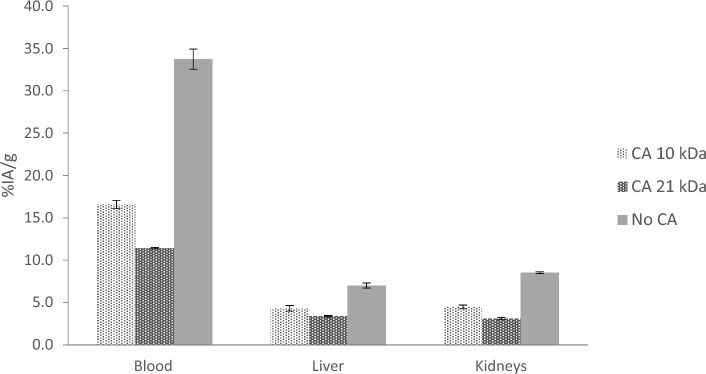
Organ uptake in mice of ^125^I-trastuzumab-transcyclooctene or fragments/conjugates thereof, following administration of tetrazine-based clearing agents (Tz-PL-CA) of two scaffold sizes, 10 and 21 kDa. The uptake in %IA/g was compared with reference animals not receiving any clearing agent (CA). The mean and standard error of the mean (SEM; error bars) are depicted

Forty-eight hours after administering the ^125^I-TMAb-TCO, the uptake of radioactivity in the liver and kidney was assessed to investigate the clearance pathway and quantify the degree of ^125^I-TMab-TCO decomposition, both in the presence and absence of CA. The blood concentration of ^125^I-TMAb-TCO in mice receiving Tz-PL-CA had dropped even further to approximately 33.8 and 49.1%, for the large and small Tz-PL-CA respectively, compared to controls (*p* < 0.0001 for both sizes), Fig. 6. The uptake of ^125^I-TMAb-TCO in the liver was approximately 51.8% and 38.6% lower in the mice injected with the large and small Tz-PL-CA respectively, compared to the control groups (*p* < 0.0001 for the large PL and *p* = 0.0023 for the small PL) while the ^125^I-TMAb-TCO uptake in the kidneys were 63.5% and 47.5% lower respectively (*p* < 0.0001 for both sizes), Fig. 6.

## Discussion

In this study, two separate types of clearing agents (CAs) based on a poly-L-lysine (PL) scaffold were synthesized and evaluated in vivo in a tumor free mouse model using two different pretargeting systems, (strept)avidin/biotin and Tetrazine/Transcyclooctene i.e. click chemistry. The general results showed that it is possible to enhance clearance of a circulating pretargeting agent from the blood using PL based PRIT strategies. The use of PL allowed for a rapid and simple synthesis of both types of clearing agents. The biotin or tetrazine residues, which are essential for the binding to the pretargeting agents, could easily be attached to the ε-amino groups of the PL via NHS ester groups. Similarly, the galactosyl groups (introduced for improved hepatic clearance) was also easily introduced using NHS ester reactions in either a single step reaction (Tz-PL-CA) or a two-step reaction (biotin-PL-CA).

The desired hepatic clearance pathway of the clearing agents in this work was predicted since the galactosyl groups on the clearing agent direct the CA-pretargeting conjugate to the Ashwell receptors in the liver. However, when investigating the (strept)avidin/biotin pretargeting system, the uptake of ^125^I-1F5-SA in the liver 68 h after injection was ~ 4 times higher in the mice receiving CA compared to controls. This indicates that the biotin-PL-CA/^125^I-1F5-SA conjugate displays a liver accumulation through an unknown mechanism. Similarly, the kidney activity uptake of ^125^I-1F5-SA was more than 2 times higher in the mice receiving CA compared to the control group. One reason that could explain the slow excretion is that the 1F5-SA is slowly decomposed into smaller fragments in the liver, which are subsequently taken up by the kidneys. For the click chemistry pretargeting system the liver uptake of the ^125^I-TMAb-TCO was instead ~ 2 times lower for the mice receiving CAs compared to controls, indicating an efficient liver metabolism of the TMAb-TCO. In line with this, the kidney uptake for the ^125^I-TMAb-TCO was ~ 2 times lower compared to controls.

Blood clearance of the pretargeting agents, 1F5-SA and TMAb-TCO, in both pretargeting systems was shown to improve considerably with the introduction of the biotin-PL-CA and the two different Tz-PL-based CAs. In the click chemistry pretargeting system, the size effect of the poly-L-lysine backbone of the CA was also explored. The larger Tz-PL-CA show more rapid clearance as compared to the smaller Tz-PL-CA, which could be due to the number of galactosyl units attached to the PL, where a larger polymer allows for a larger amount of galactose units and hence an improved clearance. That a larger amount of galactose on the CA improves the clearance is something that has been seen in several previous studies (Staudt and Herth 2023). The same explanation could also be valid for the attached number of tetrazines that is higher on the large CA, which could improve the efficiency of the click chemistry reaction to its counterpart, the TMAb-TCO.

Although the clearing agents synthesized and evaluated in this work showed good clearing abilities, there are other CAs, e.g. the biotinylated N-acetyl-galactosamine CA (NAGB), which has exhibited a more efficient blood clearance of antibody-streptavidin conjugates (Khaw et al. 2006). Additional clearing methods included antibody-PNA conjugates (Myrhammar et al. 2020), the use of dextrans (Green et al. 2016; Green et al. 2018), and the utilization of masking agents (Staudt and Herth 2023). Unlike clearing agents, which direct the pretargeting agent to the liver or kidneys, masking agents serve to shield the biorthogonal section of the pretargeting agent within the blood, preventing their binding to the effectors (Meyer et al. 2018). The benefit of the poly-L-lysine based clearing agents studied herein lies in the very simple synthesis and the fact that the backbone is available in a large variety of sizes. Taken into account, both these features make it possible to optimize poly-L-lysine based clearing agents using a number of different parameters such as the total amount and specific concentration of biotin/tetrazine and galactosyl groups attached. This warrants for further investigations of the clearing agent effect using a full pretargeting system in an in vivo tumor model, including investigating any potential tumor accumulation of the clearing agent itself.

## Conclusions

The findings from this proof-of-concept study show that poly-L-lysine based clearing agents improve blood clearence of the corresponding pretargeting agents in two different pretargeting systems; the (strept)avidin/biotin system utilizing the modified CD20 mAb 1F5 as pretargeting agent and the Tetrazine/Transcyclooctene (Tz/TCO) system using TCO-modified HER2 mAb Trastuzumab as pretargeting agent. It was also shown that a larger clearing agent (21 kDa PL scaffold) in the Tz/TCO system induced a faster clearence than its corresponding smaller version (10 kDa PL scaffold) and that in this system there was no observed liver or kidney accumulation. This study show that poly-L-lysine-based clearing agents are simple to synthesize and have good clearing properties. They show promise for further improvements in PRIT applications through optimization of e.g. the poly-L-lysine chain length and substitution rate.

## Abbreviations

CA: Clearing agent
mAb: Monoclonal Antibody
PL: Poly-L-lysine
PRIT: Pretargeted radioimmunoterapy
RIT: Radioimmunotherapy
TMAb: Trastuzumab
Tz: Tetrazine
TCO: Trans-cyclooctene
SA: Streptavidin
